# Interaction between Poly(ADP-ribose) polymerase-1 and α-synuclein pathology in Parkinson’s disease

**DOI:** 10.3389/fnins.2026.1774158

**Published:** 2026-04-13

**Authors:** Peng Zhang, Qinghua Li, Xiaojun Diao, Sihao Zeng

**Affiliations:** 1 Guilin Medical University, Guilin, Guangxi, China; 2 Laboratory of Guangxi Clinical Research Center for Neurological Diseases, Guilin, Guangxi, China; 3 Laboratory of Guangxi Zhuang Autonomous Region Clinical Translational Engineering Research Center for Digital Medicine, Guilin Medical University, Guilin, Guangxi, China; 4 Laboratory of Big Data Intelligent Cloud Management for Neurological Diseases of Guangxi, Guilin, Guangxi, China; 5 Department of Neurology, Shanghai General Hospital, Shanghai Jiao Tong University School of Medicine, Shanghai, China

**Keywords:** neurodegeneration, oxidative stress, Parkinson’s disease, PARP-1, Poly(ADP-ribose), Poly(ADP-Ribose) Polymerase-1, α-Synuclein

## Abstract

Poly(ADP-ribose) polymerase-1 (PARP-1) activation and α-synuclein (α-syn) aggregation are neuropathological hallmarks of Parkinson’s disease. This review aims to summarize the extensive interplay between these two factors. PARP-1 induces conformational changes in α-syn through structural reorganization mediated by poly(ADP-ribose) (PAR). Stress conditions resulting from PARP-1 overactivation are involved in the post-transcriptional regulation of α-syn. Oxidative and nitrative stress triggered by PARP-1 overactivation participate in the post-translational modifications of α-syn. PAR also contributes to α-syn degradation pathways, thereby influencing α-syn levels. Conversely, α-syn indirectly promotes PARP-1–dependent cell death via reactive oxygen species (ROS), suggesting a possible link through cell death pathways. These findings indicate that intracellular PARP-1, its metabolic products, and α-syn are closely associated, leading to dopaminergic neuronal vulnerability and potentially creating a vicious cycle of toxicity in PD pathology.

## Highlights

- A direct structural interaction between PAR and α-synuclein, followed by structural reorganization, induces conformational changes in α-syn.
- Stress states driven by PARP-1 hyperactivation are involved in the post-transcriptional regulation of α-synuclein.
- Oxidative/nitrative stress caused by PARP-1 hyperactivation contributes to the post-translational modifications of α-synuclein.
- PARP-1 modulates proteolytic degradation pathways, thereby regulating α-synuclein protein levels.
- α-Synuclein regulates PARP1 activity by generating reactive oxygen species (ROS) and may thereby trigger Parthanatos through the AIF/MIF pathway.

## Introduction

As a common neurodegenerative disorder affecting the central nervous system, Parkinson’s disease (PD) shows a significantly higher incidence in older populations (Ye et al., 2023). PD clinically manifests primarily through motor symptoms, such as resting tremor, bradykinesia, rigidity, and postural instability. Additionally, a range of non-motor symptoms, including depression, hyposmia, sleep disturbances, and autonomic dysfunction, frequently co-occur (Bloem et al., 2021). These symptoms significantly impair patients’ quality of life and social functioning, and progressively worsen as the disease advances. Current clinical treatment mainly relies on symptomatic interventions–such as levodopa, dopamine receptor agonists, and deep brain stimulation–which can partially improve motor symptoms but fail to delay or halt the progression of neurodegeneration; moreover, long-term use is frequently associated with motor complications and diminished therapeutic efficacy (Church, 2021). Our understanding of PD’s etiological factors has advanced considerably, with genetic predisposition, environmental insults, and aging recognized as major contributors to its complex pathogenesis (Li and Cai, 2016). The accumulation of Lewy bodies, primarily composed of aggregated and misfolded α-syn, represents a hallmark neuropathological feature of PD (Calabresi et al., 2023). α-syn is a 140-amino acid protein predominantly found within the presynaptic terminals of the central nervous system, where it primarily exists as a monomer in dynamic equilibrium. Physiologically, α-syn plays a key role in regulating the release of neurotransmitters–such as dopamine–from synaptic vesicles (Marino et al., 2022). However, α-syn aggregates can induce mitochondrial defects, compromise the function of proteasomes and lysosomes, and induce endoplasmic reticulum stress, and damage synaptic membranes (Paleologou and El-Agnaf, 2012). Its extracellular spread is mediated through mechanisms such as exosome encapsulation, direct transfer via tunneling nanotubes, free diffusion with endocytic uptake, and cell–cell contact-dependent transmission, collectively contributing to the prion-like propagation of pathological protein and the progression of neurodegeneration (Dieriks et al., 2017).

Poly(ADP-ribose) polymerase-1 is a nuclear enzyme primarily responsible for detecting DNA damage and catalyzing poly(ADP-ribosyl)ation (PARylation) within the cell nucleus (Bürkle et al., 2004). Under conditions of severe DNA damage, the overactivation of PARP-1 results in the substantial depletion of both NAD^+^ and ATP, resulting in cellular energy depletion. The overproduction of PAR polymers induces the mitochondrial release of apoptosis-inducing factor (AIF) and its cofactor, macrophage migration inhibitory factor (MIF). Subsequently, their translocation to the nucleus initiates a specific type of programmed cell death referred to as Parthanatos (Xu et al., 2023). Clinical investigations further reveal that dopaminergic neurons in the substantia nigra of PD brains exhibit markedly elevated PARP-1 activation (Kam et al., 2018) accompanied by extensive PAR polymer accumulation (Wang et al., 2003), indicating that abnormal PARP-1 expression, together with α-syn aggregation, may form a potential pathological mechanism. Considering the structural interplay between PARP-1 and α-syn, as well as emerging evidence of their functional regulation, this review focuses on recent advances in understanding the interactions between these two molecules–from direct structural associations to PARP-1-mediated regulation of α-syn synthesis and degradation, and the influence of α-syn on PARP-1 homeostasis. A deeper understanding of PARP-1’s role in neuronal α-syn pathology may provide an important foundation for evaluating the feasibility of targeting PARP-1–α-syn interactions as a therapeutic strategy in PD.

## Direct structural association between PARP-1 and α-syn

Evidence suggests that PAR interacts directly with α-syn, significantly accelerating its fibrillation kinetics and promoting the formation of highly pathogenic and neurotoxic fibrillar strains (Kam et al., 2018). At the atomic level, the binding between PAR and lysine (Lys)/arginine (Arg) side chains in the N-terminus of α-syn is primarily driven by electrostatic attraction between negatively charged phosphate groups and the positively charged amine/guanidinium groups, as demonstrated by NMR and biochemical analysis (Kam et al., 2018). Kam et al. (2018) using thioflavin T fluorescence assays, further confirmed the formation of the α-syn–PAR complex and demonstrated that low concentrations of PAR (5 nM) significantly shortened the fibrillation phase of α-syn, thereby enhancing its neurotoxicity. Intriguingly, approximately 20% of α-syn detected in the brains of mice injected with α-syn preformed fibrils (PFFs) was found to be PAR-bound (Kam et al., 2018). Histological analyses in C57BL/6 wild-type and PARP-1 knockout mouse models revealed that, as opposed to the group treated solely with α-syn PFFs, injection with PAR–α-syn PFFs induced a marked loss of dopaminergic neurons 3 months post-injection (Kam et al., 2018).

These findings indicate that direct binding and subsequent structural rearrangement largely lock α-syn into conformations that may hold physiological or pathological significance. To date, the structural and biochemical properties of the PAR–α-syn complex–as well as its functional role in disease–remain to be elucidated, owing to its high conformational heterogeneity, variable binding sites and stoichiometry, and pronounced hydration effects.

## Transcriptional and post-transcriptional regulation of α-syn by PARP-1

At the transcriptional level, PARP-1 exerts regulatory effects by binding to specific cis-regulatory elements. Chiba-Falek et al. (2005) demonstrated that PARP-1 can specifically bind to the NACP-Rep1 polymorphism site upstream of the *SNCA* gene, thereby directly driving the transcription of α-syn. This discovery provides direct evidence for the role of PARP-1 in the pathology of α-syn. Additionally, PARP-1 can also regulate various transcription factor complexes, such as NF-κB (Ting et al., 2025) and AP-1 (Wunsch et al., 2023), which may indirectly affect the expression of the *SNCA* gene. Cheli et al. (2010) demonstrated that PARP-1 can modify the DNA binding ability of transcription factors such as Sp1 through PARylation, and this mechanism is also expected to act on the transcriptional regulation of the *SNCA* promoter region. Moreover, PARP-1 regulates HIF-1α accumulation during nitric oxide and oxidative stress pathways (Martínez-Romero et al., 2008), highlighting its role in coordinating transcriptional responses to stress that may impact α-syn homeostasis.

Regarding post-transcriptional regulation, the evidence remains largely inferential. While α-syn expression is known to be tightly regulated by miR-7 (Doxakis, 2010), it has been suggested that complex ceRNA networks might further modulate this axis (Sang et al., 2018). Additionally, recent findings indicate that PARP-1 activation can induce the PARylation of LDH-B, contributing to metabolic dysfunction and neuronal death (Chen et al., 2024). However, whether these PARP-1-mediated metabolic or epigenetic changes directly govern the miRNA-dependent regulation of α-syn remains speculative and warrants further experimental validation. Building on findings from other proteinopathy models, PAR-mediated stress granules have been proposed as potential sequestration sites for various transcripts (Duan et al., 2019; Wolozin and Ivanov, 2019). By extension, it is hypothesized that *SNCA* mRNA might be similarly sequestered, with its post-transcriptional fate modulated by PARP-1 enzymatic activity and PAR turnover. However, direct experimental evidence confirming this specific regulatory axis for α-syn in Parkinson’s disease is currently lacking, and this mechanism remains a theoretical model.

## Post-translational modifications of α-syn mediated by PARP-1

Post-translational modifications (PTMs) of α-syn play critical roles in regulating its conformation, aggregation properties, and neurotoxicity. Numerous studies have demonstrated that these modifications alter the physicochemical properties of α-syn, thereby influencing its physiological functions as well as its propensity to aggregate under pathological conditions. Among PTMs, phosphorylation stands out as the most widely investigated. Among these, phosphorylation at Ser129 is widely recognized as a hallmark of pathological inclusions such as Lewy bodies, and is markedly upregulated in synucleinopathies including Parkinson’s disease (Arawaka et al., 2017). Phosphorylation at other sites–including Ser87 (Paleologou et al., 2010), Tyr125 (Chen et al., 2009), Tyr133 (Wen et al., 2018), and Tyr136 (Sano et al., 2021)–has also been shown to alter α-syn’s conformation and the speed of its aggregation, which in turn influences its ability to form fibrils. Ubiquitination (Ho and Wing, 2024), commonly occurring at Lys12, Lys21, and Lys23, predominantly regulates the proteasomal degradation of α-syn, which in turn affects its homeostasis within the cell. SUMOylation, such as at Lys96 and Lys102, has been shown to modulate α-syn’s aggregation propensity and its nuclear distribution (Kim et al., 2011). N-terminal acetylation, a common constitutive modification, stabilizes the N-terminal helical structure of α-syn, enhances its interaction with lipid membranes, and suppresses aberrant aggregation (Bell and Vendruscolo, 2021). Conversely, oxidative and nitrative modifications–such as nitration of Tyr39–typically promote α-syn oligomerization and fibril formation, while altering its interactions with biological membranes (Hassanzadeh et al., 2024). Advanced glycation end products (AGEs), for example, modifications induced by methylglyoxal (MGO), can covalently crosslink α-syn, increasing its stability and toxicity (Vicente Miranda et al., 2017). Lipid peroxidation products such as 4-hydroxy-2-non-enal (4-HNE) can covalently modify α-syn, enhancing its membrane pore-forming activity and exacerbating neurotoxicity (Andersen et al., 2021).

As mentioned earlier, the non-covalent binding of PAR to α-syn significantly accelerates its pathological aggregation process. Recent advancements in mass spectrometry-based proteomics and structural biology have highlighted that α-syn possesses a complex PTM landscape, where the interplay among distinct modification sites profoundly influences its structural stability and conformational transitions (Hassanzadeh et al., 2024). Within this conceptual framework, the PARP-1-mediated PAR signaling pathway–encompassing the accumulation of PAR chains and their interactions with proteins–represents a crucial regulatory dimension. While the direct “modification code” for α-syn in this context remains to be fully elucidated, it is plausible that PAR signaling may jointly reshape the metabolic fate and neurotoxic potential of α-syn through potential cross-talk with other PTMs, such as phosphorylation and ubiquitination.

This review will specifically highlight the roles of PARP-1 in mediating *in vivo* nitration and phosphorylation of α-syn, as well as structural and functional changes in α-syn associated with PARP-1–induced oxidative stress.

## Nitrative modification of α-syn mediated by PARP-1

Poly(ADP-ribose) polymerase-1 is not a “nitrating enzyme” in the conventional sense; rather, its involvement in the nitrative modification of α-syn is mediated through an indirect, multi-step cascade that depends on its enzymatic activity.

As a nuclear DNA repair enzyme, PARP-1 can become hyperactivated under genotoxic stress, thereby promoting inflammatory signaling via the NF-κB pathway and upregulating the expression of inducible nitric oxide synthase (iNOS) (Oliver et al., 1999). The progressive loss of dopamine-producing neurons within the substantia nigra is a primary pathological feature of PD, accompanied by a reduction in dopamine levels–a pathological decline closely associated with inflammation and oxidative stress. Studies have reported that PD models frequently display augmented endoplasmic reticulum (ER) stress and increased levels of inflammatory cytokines such as tumor necrosis factor-α (TNF-α),which can induce iNOS gene expression and increase its mRNA abundance (More and Choi, 2017). This elevation in iNOS mRNA corresponds to enhanced protein expression, resulting in a sustained increase in nitric oxide (NO) production and exacerbation of neuronal injury. These observations are consistent with findings that PARP-1 activation leads to higher iNOS protein and mRNA levels. Furthermore, activated PARP-1 can interact with regulatory regions in the iNOS promoter, thereby promoting its transcription and protein expression, which provides a molecular basis for subsequent nitrative modification of α-syn (Yu et al., 2006). Being an inducible synthase, iNOS exhibits very low or undetectable expression levels in a normal physiological state; however, under the regulatory influence of PARP-1, its activity can be markedly increased (Naura et al., 2009). In this state, iNOS efficiently and persistently synthesizes large amounts of NO using L-arginine as a substrate (Naura et al., 2009). INOS is a key enzyme in generating excess NO, which reacts with superoxide anion to yield peroxynitrite (ONOO)–one of the principal mediators of protein tyrosine nitration (Pazzaglia and Pioli, 2019). Therefore, alterations in PARP-1 activity may modulate α-syn nitration levels by regulating iNOS expression.

## Phosphorylation of α-syn mediated by PARP-1

In biological systems, phosphorylation is one of the most prevalent forms of post-translational modification, involving the transfer of a phosphate group to specific amino acid residues (such as serine, threonine, or tyrosine) by kinases (Seok, 2021). Under neuropathological circumstances, phosphorylation of α-syn is considered a key factor driving its abnormal aggregation and onset of PD and other synucleinopathies, with Ser129 being the predominant site (Zhang et al., 2015). Studies have shown that the proportion of Ser129-phosphorylated α-syn in normal brain tissue is less than 5%, whereas in pathological bodies it exceeds 90%, markedly enhancing α-syn oligomerization and neurotoxicity (Ramalingam and Dettmer, 2023; Parra-Rivas et al., 2023). PAR molecules have been observed to interact with highly phosphorylated α-syn, a phenomenon documented both in PD-related transgenic mouse models and in the brain tissue of PD patients (Puentes et al., 2021).

Poly(ADP-ribose) polymerase-1 might play a direct role in regulating α-syn phosphorylation. Under pathological conditions such as oxidative stress, hyperactivation of PARP-1 significantly promotes the accumulation of α-syn (Kam et al., 2018). Furthermore, PAR chains generated by PARP-1 induce pathogenic phosphorylation of the protein, thereby accelerating the formation of neurotoxic aggregates (Puentes et al., 2021). These research results indicate that the pathological environment formed by the excessive activation of PARP-1 promotes these specific PTMs of proteins. These findings suggest that PAR, generated via PARP-1 overactivation, serves as a critical regulator in the phosphorylation of α-syn.

## Impact of PARP-1 on α-syn degradation–Parthanatos-mediated clearance

In the pathological process of neurodegenerative diseases–particularly PD–Parthanatos is recognized as a major mechanism mediating the loss of dopaminergic neurons. Synthetic α-syn PFFs have been shown to induce neuronal death in primary cortical neurons by triggering the Parthanatos pathway, indicating that Parthanatos plays a critical role in α-syn–mediated neurotoxicity (Park et al., 2022; Wang and Ge, 2020). In PD mouse models, each of the PARP-1 inhibitors (Mao et al., 2020; Outeiro et al., 2007; Kam et al., 2018) and genetic deletion of PARP-1 (Kam et al., 2018) have been demonstrated to effectively block the toxicity of pathological α-syn. Interestingly, activation of Parthanatos can further enhance the neurotoxicity of pathological α-syn, establishing a positive feedback loop (Kam et al., 2018). PAR directly accelerates fibrillation kinetics and generates more neurotoxic forms of α-syn (Kam et al., 2018). This cascade results in a feedforward cycle in which PARP-1 hyperactivation elevates PAR levels, thereby increasing α-syn toxicity and further amplifying subsequent PARP-1 activation.

## Degradation of α-synuclein via the ubiquitin–proteasome system and the autophagy–lysosome pathway

The clearance of α-syn depends primarily on the ubiquitin–proteasome system (UPS) and the autophagy–lysosome pathway (ALP) (Sharma et al., 2025). Ubiquitin is essential for maintaining cellular protein homeostasis and regulating signaling cascades. In neurodegenerative diseases, functional impairment of ubiquitin drastically reduces the cell’s capacity to eliminate abnormal proteins via UPS, thereby increasing neuronal susceptibility to aggregated proteins and their associated toxicity. Evidence suggests that PARP-1–mediated PARylation can inhibit the ubiquitination of certain target proteins by specific E3 ubiquitin ligases–an important mechanism in modulating protein degradation and signal transduction (Zhu et al., 2023). PARP-1 has been found to interact directly with the RBR-type E3 ubiquitin ligase RNF144A, which promotes PARP-1 self-ubiquitination and proteasome-mediated degradation (Zhang et al., 2017), indicating that E3 ligases can also regulate PARP-1 stability in reverse. Furthermore, in the process of vascular calcification, the Cbl-b E3 ligase activates PARP-1 via NEDD8-dependent modification. Collectively, these findings suggest the existence of a complex reciprocal regulatory network between E3 ligases and PARP-1. In PD, α-syn is first ubiquitinated by E3 ubiquitin ligases and conjugated via K48-linked polyubiquitin chains, which are ultimately eliminated through the 26S proteasome pathway (Buneeva and Medvedev, 2022; Ho and Wing, 2024). Thus, we propose that PARP-1 hyperactivation may interfere with the ubiquitination process of certain E3 ligase substrates–either through PARylation or direct physical interaction–thereby impairing UPS-mediated α-syn clearance and promoting its intracellular accumulation and aggregation.

Autophagy is essential for maintaining cellular homeostasis and genomic integrity. Dysfunctional autophagy in neurodegenerative diseases increases the vulnerability of neurons to aggregated proteins, organelle dysfunction and oxidative damage (Jang et al., 2020). PARP-1 modulates autophagy through multiple mechanisms. It can directly PARylate autophagy-related proteins, such as AMPKα, to regulate their activities (Rodríguez-Vargas et al., 2016). Additionally, the hyperactivation of PARP-1 depletes cellular NAD^+^ pools, which in turn suppresses NAD^+^-dependent SIRT1 and indirectly impairs autophagic flux (Jang et al., 2020). Excessive activation of PARP-1 leads to massive consumption of intracellular NAD^+^ reserves, thereby reducing the activity of the NAD^+^-dependent deacetylase SIRT1 and ultimately lowering autophagy levels (Cantó and Auwerx, 2011; Ke et al., 2019). Hyperactivated PARP-1 can also suppress autophagy gene expression via PARylation of the transcription factor TFEB, downregulating autophagy-related factors such as LC3-II. Inhibition of PARP-1 has been shown to stimulate autophagic activity and promote α-syn degradation (Zhang et al., 2023; Xu et al., 2020; Sun et al., 2015). Mao and Zhang (2022), reported that PARP-1 inhibitors enhance autophagy, thereby facilitating α-syn clearance. These findings indicate that PARP-1 modulates α-syn removal efficiency by regulating the autophagy pathway.

## Genetic factors influencing α-syn regulation by PARP-1

Multiple genetic factors may directly or indirectly disrupt α-syn degradation mechanisms. Genetic aberrations in the *SNCA* gene, including but not limited to mutations and copy number variations, are known to cause a substantial elevation in α-syn protein expression. Studies have shown that PARP-1 modulates α-syn transcription by binding to the upstream NACP-Rep1 polymorphic site of the *SNCA* gene (Chiba-Falek et al., 2005). Experimental evidence indicates that inhibition of PARP-1 catalytic activity significantly elevates endogenous *SNCA* mRNA levels in SH-SY5Y cells (Chiba-Falek et al., 2005), suggesting that PARP-1 negatively regulates *SNCA* transcription through its enzymatic function. In addition, PARP-1 can regulate the transcription of multiple key genes involved in cellular function (Luo and Kraus, 2012). PARP-1 participates in chromatin remodeling and promotes the activation of the c-Myc transcription factor, thereby regulating cell proliferation and differentiation (Simbulan-Rosenthal et al., 2003; Zong et al., 2022). This indicates that PARP-1 also plays a crucial role in cell cycle reactivation and the expression of early response genes. Although its main characteristics are in the context of tumor occurrence and development, this PARP-1-dependent cell cycle reactivation has also been increasingly associated with abnormal re-entry of post-mitotic neurons into the cell cycle, which is a potential pathological mechanism in neurodegenerative diseases (including Parkinson’s disease) (Höglinger et al., 2007).

## Disruption of PARP-1 homeostasis by α-syn

Aggregated α-syn exerts detrimental effects on mitochondrial function, elicits oxidative stress, and induces DNA damage, ultimately resulting in the activation of PARP-1. Interaction between α-syn and mitochondrial respiratory chain complex I leads to the formation of pores in the mitochondrial membrane, resulting in membrane potential loss and impaired oxidative phosphorylation, ultimately reducing ATP production efficiency. This dysfunction causes mitochondria to generate excessive ROS, thereby promoting oxidative stress (Xiang, 2025; Dening et al., 2022; Geibl et al., 2024). In addition, oligomeric α-syn exacerbates mitochondrial injury and oxidative/nitrative stress by downregulating Parkin expression (Domingues et al., 2022). Enhanced oxidative stress in turn results in DNA damage–an important signal for PARP-1 activation. Hyperactivation of PARP-1 consumes large amounts of NAD^+^ and ATP, precipitating an energy crisis and promoting Parthanatos (Xiang, 2025). Thus, α-syn indirectly promotes PARP-1 activation by inducing mitochondrial dysfunction and oxidative stress. Beyond its cytoplasmic presence, α-syn can also translocate into the nucleus and directly bind DNA (Kontopoulos et al., 2006; Schaser et al., 2019). *In vitro* biochemical analyses have shown that both monomeric and oligomeric α-syn bind double-stranded DNA through electrostatic interactions, leading to conformational changes such as local bending, unwinding, or relaxation of supercoils (Mukherjee et al., 2021). Such direct damage increases the burden on DNA repair systems and persistently activates PARP-1. In its capacity as a damage-associated molecular pattern, oligomeric α-syn is specifically bound by Toll-like receptors 2 and 4 (TLR2/4) expressed on microglia, which in turn triggers the downstream NF-κB signaling cascade (Li et al., 2021; Jurcau et al., 2023). This pathway may further induce transcriptional upregulation and activation of PARP-1. Despite the clear pathological synergy, definitive molecular evidence of α-syn physically binding to and directly activating PARP-1 remains elusive, and we propose searching for direct molecular bridges as an important research direction for this field in the future.

## Perspectives

**FIGURE 1:**
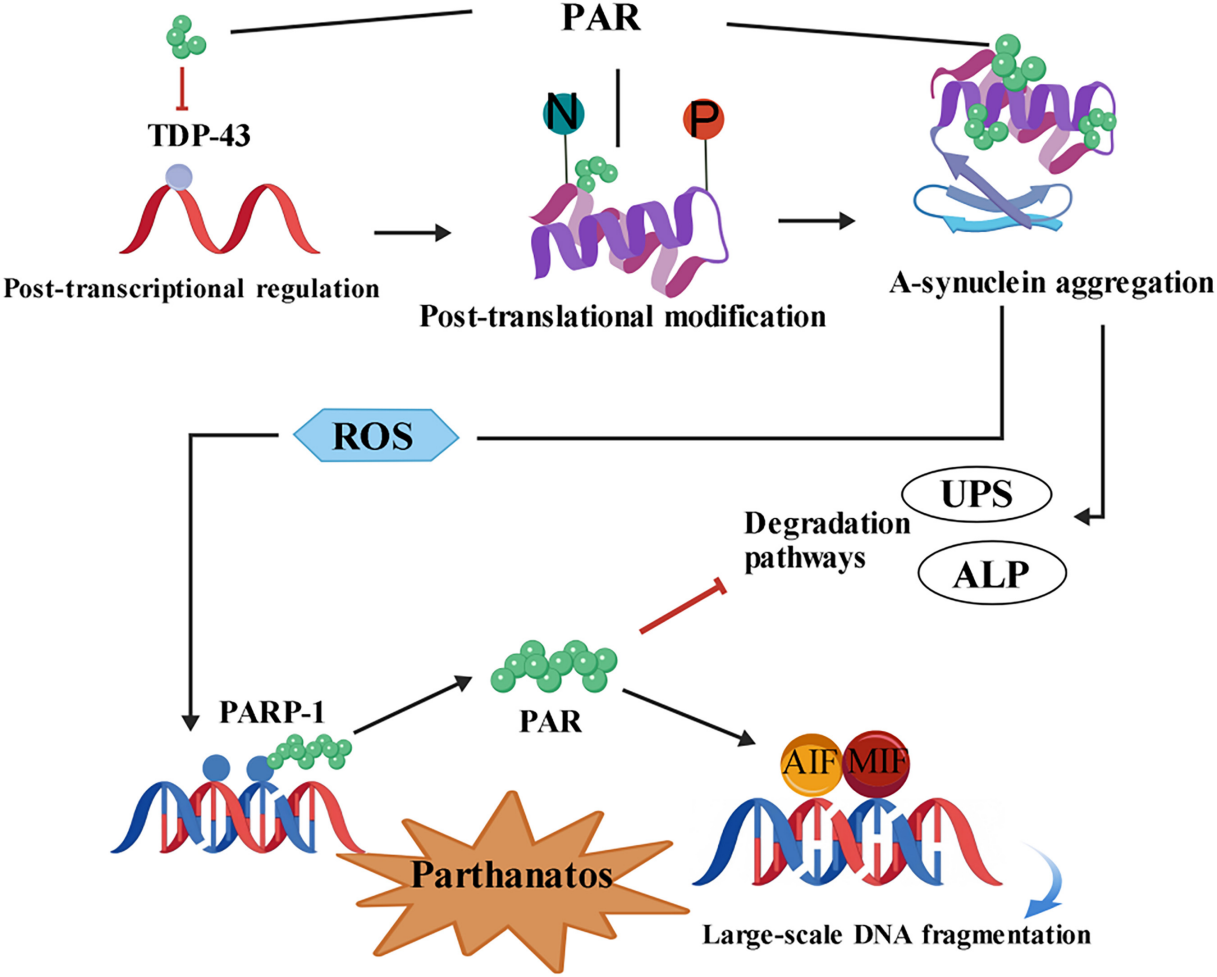
Interactions between PARP-1 and α-synuclein pathology in Parkinson’s disease. Upon PARP-1 overexpression, PAR inhibits the ability of the stress-granule protein TDP-43 to protect mRNA. α-syn binds PAR and undergoes conformational changes, while post-translational modifications induce PARP-1-associated nitrative/oxidative stress–such as nitration (N) or phosphorylation (P)–leading to α-syn aggregation. Under physiological conditions, α-syn is predominantly degraded via the ubiquitin–proteasome system (UPS) and the autophagy–lysosome pathway (ALP). Intracellular PAR can directly inhibit these degradation pathways, thereby contributing to α-syn accumulation. α-Syn enhances reactive oxygen species (ROS) production, triggering DNA damage and causing hyperactivation of PARP-1, which generates large amounts of PAR. The accumulated PAR subsequently activates the AIF-MIF pathway, leading to Parthanatos cell death.

α-synuclein and PARP-1 are both actively involved in the pathological process of PD, forming a self-perpetuating vicious cycle. Within neurons, PARP-1 interacts with α-syn, contributing to the heightened vulnerability of dopaminergic neurons in PD (Figure 1). These findings suggest that targeting PARP-1, α-syn aggregation, and their pathological interplay may represent a promising therapeutic strategy for PD. There are still some key knowledge gaps. Although there is indirect evidence suggesting that α-syn promotes PARP-1 activation through reactive oxygen species, whether α-syn can directly interact with PARP-1 in the cell nucleus to regulate its catalytic activity remains an unresolved issue. Future research should focus on investigating whether other members of the PARP family are involved in the pathological process of α-syn, which may reveal redundant or synergistic pathways. From a translational research perspective, some challenges must be addressed. Although PARP inhibitors are currently applied in oncology, their long-term safety for chronic neurodegenerative diseases still requires strict validation, especially regarding their impact on DNA repair in non-dividing neurons. Additionally, determining reliable biomarkers that can reflect the activation state of PARP-1 and the aggregation of α-syn in patients will be crucial for patient classification and evaluating treatment efficacy in future clinical trials. Targeting the PARP-1/α-syn axis is an effective strategy, but its success depends on achieving high blood-brain barrier permeability and minimizing non-targeted systemic toxicity.
